# Electrospinning Preparation of Silk Fibroin/Titanium-Based Photocatalytic Fiber Membrane for Bacteria Disinfection in Wastewater

**DOI:** 10.3390/polym18131632

**Published:** 2026-06-30

**Authors:** Kuo Wang, Xiaoxuan Liu, Dading Zhou, Yujun Wang, Qiansu Ma, Yingnan Yang, Na Liu

**Affiliations:** 1 Department of Biomedical Engineering, University of Chengde Medical, Chengde 067000, China; 13293245945@163.com (K.W.); heee2782@163.com (X.L.); 18353236072@163.com (D.Z.); 2 Department of Biological and Food Sciences, University of Chengde Medical, Chengde 067000, China; wangyj@cdmc.edu.cn; 3 School of Chemistry and Biological Engineering, University of Science and Technology Beijing, Beijing 100083, China; qiansuma@ustb.edu.cn; 4 Graduate School of Life and Environmental Sciences, University of Tsukuba, 1-1-1 Tennoudai, Tsukuba 305-8577, Ibaraki, Japan; yo.innan.fu@u.tsukuba.ac.jp

**Keywords:** electrospinning, silk fibroin, SF/PAgT fiber membrane, visible light photocatalysis, antibacterial activity, Gram +ve/−ve, antibacterial mechanism, wastewater

## Abstract

Most traditional photocatalysts exist in powder form and have the disadvantage of being difficult to recycle and causing secondary pollution to the environment after use. To overcome this drawback, this study combined natural biopolymer (silk fibroin (SF)) with a previously developed titanium-based photocatalytic material P/Ag/Ag_2_O/Ag_3_PO_4_/TiO_2_ (PAgT) and fabricated a novel SF/PAgT fiber membrane via electrospinning. During the synthesis process, through adjusting the mass concentration of the PAgT dopant (0–0.30 g/mL), a series of photocatalytic fiber membranes were prepared. The morphology and structure of the as-prepared membranes were characterized by various analytical methods, including scanning electron microscopy (SEM), X-ray diffraction (XRD), Fourier transform infrared (FT-IR), contact angle (CA) and thermogravimetric analysis (TGA). The SEM images confirmed that the SF/PAgT composite membrane possessed a protrusive and spindle-shaped structure. FT-IR results verified that the primary structure of SF in all the as-prepared SF/PAgT membranes belonged to the Silk II type. The binding of SF with the PAgT photocatalyst did not disrupt the chemical structure and original properties of SF. Moreover, the XRD and CA measurements indicated that the SF/PAgT-4 fiber membrane exhibited the stronger diffraction peaks of anatase TiO_2_ crystal structure and enhanced hydrophilicity. The experimental results clarified that the PAgT photocatalyst was successfully loaded onto the SF fiber membrane by electrospinning. To evaluate the performance of the developed visible-light-driven photocatalytic fiber membranes, Gram-negative *Escherichia coli* (*E. coli*) and Gram-positive *Staphylococcus aureus* (*S. aureus*) were selected as representative bacteria strains. The results demonstrated that SF/PAgT-4 exhibited the optimal antibacterial activity and can completely inactivate 10^7^ CFU/mL of *E. coli* and *S. aureus* within just 30 min and 60 min treatment, respectively, indicating the optimal doping mass concentration of PAgT during the synthesis process was 0.20 g/mL. Furthermore, the scavenger study proved that during the photocatalytic disinfection process by SF/PAgT-4, all three radicals, including ·OH, h^+^ and ·O_2_^−^, participated in the current photocatalytic disinfection system. They were capable of attacking the bacterial cells, causing the cell membrane injury, thereby leading to the intracellular component leakage and inducing extensive bacterial inactivation. Hence, by virtue of its excellent recyclability (during five cycles) and thermal stability (below 250 °C), the developed SF/PAgT-4 fiber membrane holds immense potential for highly efficient and sustainable utilization in practical water treatment applications.

## 1. Introduction

With the global population growth and rapid industrial development, the shortage of clean drinking water has become one of the most urgent challenges threatening human health [1]. According to statistics, approximately 1.8 million people die each year due to diseases caused by water-borne pathogenic microorganisms [2]. Traditional disinfection methods, such as ultraviolet irradiation [3], chlorination and ozone oxidation [4]—despite having alleviated the problem of microbial contamination in water to some extent—often encounter bottlenecks such as high energy consumption and the generation of toxic or carcinogenic by-products [5]. Therefore, the development of a stable, safe and environmentally friendly efficient antibacterial strategy is necessary. Compared with the limitations of traditional water disinfection technologies, the new photocatalytic technology has attracted much attention due to its ability of environmental protection, long-term stability, and nontoxicity [6]. The core mechanism of photocatalysis lies in that when the material is exposed to light with energy greater than its band gap, it will generate e^−^-h^+^ pairs. Then the photogenerated e^−^-h^+^ will react with water and oxygen molecules to produce reactive oxygen species (ROS), such as hydroxyl radicals (·OH) and superoxide anions (⋅O_2_^−^) [7,8]. These ROS with strong oxidizing ability can directly oxidize the cell walls, proteins, and genetic materials of bacteria, effectively blocking their reproduction at the source [9,10]. Moreover, the photocatalytic process does not produce secondary pollutants, which is in line with the modern green concept of sustainable development [11].

Today, various photocatalytic nanomaterials, such as zinc sulfide (ZnS), titanium dioxide (TiO_2_), cadmium sulfide (CdS), tungsten trioxide (WO_3_) and bismuth vanadate (Bi_2_VO_4_), have been developed for pollutant removal and disinfection in wastewater treatment [8]. Among them, TiO_2_ is one of the most widely used materials in this field due to its excellent stability, energy efficiency, low cost, and biocompatibility. However, TiO_2_ nanoparticles have a wide bandgap (anatase: 3.2 eV), which limits their application. It typically exhibits photocatalytic activity only under UV, which accounts for only 4–5% of total solar radiation [12]. This means that TiO_2_ nanoparticles have a very low utilization rate of sunlight. The second disadvantage of pure TiO_2_ is the high recombination efficiency of e^−^ and h^+^, which also leads to a reduction in its photocatalytic activity. Therefore, to overcome the aforementioned drawbacks, a significant amount of research studies have been devoted to the modification of TiO_2_ nanoparticles.

In order to solve the problem of low solar light utilization efficiency and high e^−^-h^+^ recombination efficiency of pure TiO_2_, scientists have adopted a series of modification strategies to develop more efficient photocatalytic materials, such as metal doping, non-metal doping, or hybridization with narrow bandgap semiconductors. For example, Sultana et al. doped manganese (Mn) metal atoms and boron (B) non-metal atoms into TiO_2_, enabling the material to degrade Rhodamine B (Rh B) under visible light irradiation [13]. Similarly, Salzano et al. modified TiO_2_ with N and Ag, so that the material had sterilization performance under visible light [14]. In addition, some reports modified TiO_2_ with narrow band gap semiconductors. Rashid et al. doped Ag_3_PO_4_ into TiO_2_, and the modified material could achieve 92.5% degradation of 2-chlorophenol within 120 min [14]. Therefore, an effective way to enhance the photocatalytic performance of TiO_2_ in the visible light region is to dope it with non-metallic and metallic elements. This enhances visible-light activity by forming new band structures or suppressing the recombination of photo-generated e^−^-h^+^ pairs, thereby improving the quantum efficiency. Compared with UV-light-driven photocatalysis, visible-light-driven photocatalysis can utilize the dominant visible region of the solar spectrum, avoiding dependence on high-energy UV irradiation. Such a system could improve solar energy utilization, cut operating energy costs, and be more promising for practical large-scale environmental applications under natural sunlight. As such, based on the aforementioned methods, in our previous study, a novel visible-light-driven photocatalyst P/Ag/Ag_2_O/Ag_3_PO_4_/TiO_2_ (PAgT) had been successfully developed via a sol-gel/hydrothermal two-step method combined with the co-doping of non-metallic, metallic, and narrow-bandgap materials [15]. It was found that the newly developed PAgT can fully degrade 50 mL of 2 mg/L Rh B solution in 30 min and completely eliminate 15 mL of 10^7^ CFU/mL *E. coli* within 20 min. Meanwhile, a hydrogen production rate of 1326.0 mmol/g was attained for PAgT after 3 h of reaction. Previous studies have demonstrated that the developed PAgT material exhibits excellent performance under visible light in the decomposition of organic dye (Rh B), bacterial inactivation, and water splitting for hydrogen production [15,16].

Although numerous highly efficient photocatalytic materials have been developed, most exist in powder form, and many of them are nanoscale. In practical application, photocatalysts in powder form face technical bottlenecks such as easy agglomeration and difficult recovery in practical applications [17]. To address this challenge, immobilizing photocatalysts on a carrier or support can serve as an effective approach. For example, Wang et al. used expanded perlite (EP) particles as a carrier and employed the sol-gel method to synthesize the B-N-TiO_2_/EP composite. Under 3 h of visible light irradiation, this composite achieved a 94% photodegradation rate for Rh B [18]. Song et al. used negative pressure filtration technology to embed the 3D graphene oxide/carbon quantum dot/copper ferrite (G/CFQ) photocatalyst between the graphene oxide (GO) nanosheets to prepare the M-G/CFQ membrane. The M-G/CFQ membrane achieved a degradation rate of 98.4% for methylene blue (MB) within 6 h [19]. Similarly, Wang et al. used the flat-edge scraping method and phase transformation method to prepare the Zn-Cu-TiO_2_/Polyvinylidene fluoride (PVDF) membrane. Under 2 h of visible light irradiation, the Zn-Cu-TiO_2_/PVDF membrane achieved a 91.6% photodegradation rate for sulfadiazine [20]. In addition to those mentioned above, the commonly used immobilization techniques include the in-situ growth method, physical adsorption and other methods [21]. These immobilization strategies not only enable the effective separation of the catalyst from the reaction medium but also facilitate its recovery and reuse, thereby establishing an efficient and recyclable reaction microenvironment during the reaction. However, the widespread implementation of these strategies is often hampered by their substrate specificity, intricate fabrication procedures, or reliance on toxic reagents [22,23]. Furthermore, the problems of large-scale production and compatibility with industry remain significant constraints [24]. Compared to these conventional methods, electrospinning technology stands out for its ability to produce fiber membranes with continuous structures and extremely high specific surface areas [25]. Unlike traditional particle-loaded carriers, electrospinning fiber membranes form a unique three-dimensional interconnected porous network, which not only provides abundant active sites for the uniform loading of photocatalysts but also significantly enhances the physical capture ability of the membrane material for target microorganisms [26]. By precisely adjusting the spinning parameters, the fiber diameter and pore structure can be customized, thereby optimizing the light penetration depth and reaction substance diffusion efficiency. Therefore, the development of functionalized composite membranes using electrospinning technology has become one of the key paths for realizing the transition of photocatalytic antibacterial technology from the laboratory to practical industrial applications [27].

Until now, more than 100 different types of organic polymers have been successfully explored for electrospinning to directly produce nanofibers [28], such as synthetic polymers (polystyrene (PS) [29] and poly vinyl chloride (PVC) [30]), natural biopolymers (chitin [31] and silk fibroin (SF)). It is worth noting that, although synthetic polymers have been widely used in electrospinning, their non-biodegradability and potential risk of microplastic pollution were increasingly being scrutinized by the academic community. In contrast, SF as a natural biopolymer was a natural high-molecular protein mainly derived from silk. Its unique molecular structure is rich in β-sheet crystalline domains, featuring excellent spinning properties and enabling the fabrication of nanofiber matrices suitable for biomedical and environmental applications [32]. Furthermore, due to the excellent biocompatibility, biodegradability and cell adhesion properties of SF, it has been extensively studied as a coating material for biological materials [33]. SF was widely used in the textile field with its excellent mechanical properties. SF demonstrated excellent processing capacity and could be flexibly shaped into various morphological structures even at the nanoscale. For example, Zhao et al. used the SF with polyurethane (PU)/AgI to produce a membrane to remove tetracycline under visible light [34]. It could achieve efficient binding with a variety of active ingredients, which opened up a broad application space for the development of SF fiber materials with controllable structure and adjustable function. Therefore, by utilizing electrospinning technology to combine SF with the previously developed high-efficiency photocatalytic material (PAgT), the current bottleneck regarding the difficult recovery and reuse of powdered photocatalytic materials will be effectively resolved. This will hold broad prospects for application in the field of practical water treatment, and to date, no related research has been reported.

Herein, in this study, a new type of photocatalytic composite fiber membrane was developed by integrating PAgT photocatalyst powder with silk fibroin (SF) via electrospinning. By adjusting the doping mass concentration of PAgT, a series of distinct fiber membranes (SF/PAgT) were fabricated and subsequently characterized by scanning electron microscopy (SEM), X-ray diffraction (XRD), Fourier transform infrared spectroscopy (FT-IR), contact angle (CA) and thermogravimetric analysis (TGA) to examine the physicochemical properties of the prepared materials, thereby determining the optimal doping concentration of PAgT. The antibacterial effects of SF/PAgT photocatalytic membrane against different types of bacteria were investigated, including Gram-positive (Gram +ve) *Staphylococcus aureus* (*S. aureus*) and Gram-negative (Gram −ve) *Escherichia coli* (*E. coli*). Furthermore, the recyclability and thermal stability of the developed photocatalytic membrane were examined to assess its practicality. Finally, the photocatalytic mechanism of the SF/PAgT membrane was proposed to deeply understand its antibacterial process.

## 2. Materials and Methods

### 2.1. Materials

Silkworm cocoons used in this study were provided by Chengde Medical University. Nutrient broth, nutrient agar, Eosin-Methylene Blue Agar (EMB agar) and phosphate-buffered saline (PBS) for microbial culture were purchased from Huankai Biotechnology Co., Ltd. (Guangzhou, China) at biological reagent (BR) grade. TiO_2_ P25 was purchased from Evonik Industries (Essen, Germany). Tetrabutyl titanate, nitric acid (HNO_3_) and anhydrous methanol were provided by Sinopharm Group Chemical Reagent Co., Ltd. (Shanghai, China). Silver phosphate (Ag_3_PO_4_) was provided by Kemiou Chemical Reagent Co., Ltd. (Tianjin, China). Formic acid (98%), polyethylene oxide (PEO), sodium oxalate (Na_2_C_2_O_4_), isopropanol, TEMPOL (98%), anhydrous calcium chloride, silver nitrate (AgNO_3_), propidium iodide (PI, 98%), 4′,6-diamidino-2-phenylindole (DAPI, 98%) and anhydrous ethanol were purchased from Aladdin Biochemical Technology Co., Ltd. (Shanghai, China). Unless otherwise specified, all the chemicals were reagent grade.

### 2.2. Preparation of SF

The specific process of SF extraction was as follows: First, 50 g of silkworm cocoons were weighed and then placed into 10 L of boiling 0.5% Na_2_CO_3_ solution, and the mixture was boiled for 1 h. Then, the silkworm cocoons were repeatedly washed with deionized water and dried to obtain SF. Next, CaCl_2_, anhydrous ethanol and H_2_O were mixed in a molar ratio of 1:2:8. The obtained SF was added to the above solution and stirred at 60 °C until it was completely dissolved. The solution was then dialyzed continuously for three days, followed by centrifugation to remove the precipitate, and finally freeze-dried for 48 h to obtain purified SF [35].

### 2.3. Preparation of SF/PAgT Membranes by Electrospinning Method

The PAgT photocatalyst was prepared by the sol-gel/hydrothermal two-step synthesis method according to our previous published paper, and the physiochemical properties of the developed PAgT photocatalyst powder have been systematically investigated [18]. The electrospinning solution was first prepared by dissolving 0.1 g of PEO and 1.084 g of lyophilized SF powder in 8 mL of formic acid [36]. The prepared SF solution was magnetically stirred for 6 h until PEO was fully dissolved. Subsequently, the photocatalyst PAgT was dispersed into the SF solution, stirred for 10 min at a mass concentration of 0, 0.01, 0.05, 0.10, 0.20, and 0.30 g/mL, and was recorded as SF, SF/PAgT-1, SF/PAgT-2, SF/PAgT-3, SF/PAgT-4, and SF/PAgT-5, respectively. When the solution was ready, the electrospinning parameters were set, and the electrospinning process began. The spinning solution was drawn into a syringe, which was then fixed onto the syringe pump. Then, based on the previously published related studies [36], by adjusting the applied voltage, controlling the feeding rate, and changing the distance between the needle tip and the collection plate, the parameters for spinning were determined as a flow rate of 1 mL/h, a voltage of 15 kV, and a receiving distance of 15 cm. The solution ejected from the needle was collected on a flat collector covered with aluminum foil. The electrospinning process was carried out under room temperature conditions, and the ambient relative humidity was controlled to less than 70%. The total duration of the electrospinning process was approximately 1 h. After electrospinning, the fiber membranes were dried at room temperature to remove formic acid. Subsequently, the fiber membrane was immersed in anhydrous methanol to induce the formation of β-sheet, followed by drying at room temperature. In addition, the reference SF/TiO_2_ fiber membrane was also fabricated by adopting the same procedure described above for the SF/PAgT-4 membrane.

### 2.4. Characterization of Prepared Materials

The sample’s morphology was visualized on a scanning electron microscope (SEM, ZEISS, Sigma 360, Oberkochen, Germany) at an acceleration voltage of 3 kV or 15 kV, with all specimens pretreated by gold sputtering. The elemental distribution of the prepared photocatalytic fiber membranes was analyzed on an energy dispersive X-ray spectrometer (EDS, Thermo Fisher, K-Alpha, Hillsboro, OR, USA). Fourier transform infrared (FT-IR, Thermo Fisher, Nicolet iS20, USA) spectroscopy was performed to characterize the prepared SF/PAgT fiber membranes in the range of 500–4000 cm^−1^ with a resolution of 4 cm^−1^. X-ray diffraction (XRD, SmartLab SE, Tokyo, Japan) with Cu-Ka radiation was performed to examine the phase composition of the specimens produced. A scan rate of 2°/min was selected, and the scanning region of 2θ angles was determined from 10 to 80°. The contact angle (CA, SDC-100S, SINDIN Precision Instrument Co., Ltd., Dongguan, China) was used to analyze the hydrophobicity of materials. In the contact angle test, all samples were measured independently three times, and the results were expressed as the arithmetic mean of the three measurements. The thermogravimetric analysis (TGA, STA200, Hitachi High-Tech Corporation, Tokyo, Japan) was performed to obtain the TGA curves of the as-prepared fiber membranes. The heating rate of the instrument is 10 °C/min, and the test is conducted in a nitrogen atmosphere with a gas rate of 120 mL/min.

### 2.5. Photocatalytic Activity Evaluation and Cyclic Experiments

In order to evaluate the photocatalytic disinfection capability of the developed fibers, Gram −ve *E. coli* (China Medical Culture Collection Center (Bacteria) (CMCC(B)) 44102) and Gram +ve *S. aureus* (CMCC(B)26003) were selected as the model bacteria for the inactivation experiment. Both of these bacteria were obtained from the Shanghai Luwei Microbial Co., Ltd. (Shanghai, China). The preparation method of the bacterial suspension was carried out according to our previously published paper [37]. Then, the prepared bacterial suspension was resuspended in PBS to adjust the concentration to 10^7^ colony-forming units (CFU)/mL. During the photocatalytic disinfection experiment, 30 mg (2 cm × 2 cm) of the prepared SF/PAgT and SF/TiO_2_ sample was added to 15 mL of bacterial solution [22]. An LED lamp with an intensity of 200 W/m^2^ was used as light source to provide visible light (light wavelength: 400–700 nm). The SF/TiO_2_ fiber membrane with visible light was set as the positive control, and the SF/PAgT-4 fiber membrane treated in the dark was used as the negative control. The reaction solution was stirred with a magnetic stirrer throughout the experiment. Then, 1 mL of the reaction solution was withdrawn at 10 min intervals for *E. coli* groups and at 20 min intervals for *S. aureus* groups, followed by immediate dilution using PBS. Subsequently, 1 mL of the diluted *E. coli* sample was dispersed on the EMB medium for cultivation, while *S. aureus* was cultivated on the nutrient agar medium. Finally, plate counting was used to determine the number of viable cells in each sample. All experiments were carried out in triplicate, and the average value was calculated. Moreover, in order to investigate the stability of the photocatalytic fiber membrane, five consecutive tests were conducted on *E. coli* and *S. aureus* in the photocatalytic disinfection experiments, respectively. The specific steps of the cyclic experiment for photocatalytic inactivation are shown in Figure S1. After each round of tests, the SF/PAgT fiber membrane was separated from the bacterial solution and cleaned with water. Once the SF/PAgT fiber membrane was dried at room temperature, it was used in a new set of bacteria inactivation test. Photocatalytic inactivation efficiency (E) is defined as:

E = (1 − C*_t_*/C_0_) × 100%

where C_0_ (CFU/mL) is the initial bacterial concentration, and C*_t_* (CFU/mL) denotes the bacterial concentration at time *t*.

In addition, an inhibition zone test was also conducted to evaluate the antibacterial ability of the developed fibers. Before the experiment, pure SF, SF/TiO_2_, and SF/PAgT-4 fiber membranes were cut into circles with a diameter of approximately 6 mm and sterilized under ultraviolet light for 30 min. For both SF/TiO_2_ and SF/PAgT-4 samples, the weight percentage of photocatalyst in the membrane was approximately 28.6%. Then, the disposable culture dish was supplemented with 1 mL of 10^6^ CFU/mL of bacterial liquid and 9 mL of sterilized nutrient agar medium. After the medium plate had solidified, the cut circular fiber membranes were placed on the surface of a solid culture medium plate. The petri dish was exposed to an LED light source (200 W/m^2^) or kept in complete darkness for 10 min, followed by incubation at 37 °C under constant temperature and humidity for 16–24 h. Finally, the size of the inhibition zone was observed, and its diameter was measured with a ruler to determine the antibacterial effect.

### 2.6. DAPI-PI Fluorescence Staining Experiment

The cell viability after photocatalytic treatment was investigated using DAPI-PI fluorescence staining. Different experiment groups were designed, including light irradiation alone, SF/TiO_2_ with light and SF/PAgT-4 with or without light irradiation. Then, the treated *E. coli* and *S. aureus* cells were collected by centrifugation (6000 rpm, 10 min), and the supernatant was removed. Then, 100 μL of DAPI solution (50 μg/mL) was added, and the samples were incubated in the dark for 15 min. Subsequently, 100 μL of PI solution (100 μg/mL) was added, followed by another incubation in the dark for 10 min [37]. After the reaction, the samples were washed twice with PBS and observed under a fluorescence microscope (Olympus CKX53, Tokyo, Japan).

### 2.7. Radical Scavenging Experiment

To further investigate the roles of the active species generated by SF/PAgT fiber membrane in the photocatalytic inactivation of bacteria, different scavengers were added to the photocatalytic reaction system as described in Section 2.5. Specifically, TEMPOL (2 mM), isopropanol (5 mM), and Na_2_C_2_O_4_ (0.5 mM) were used to quench superoxide radicals (·O_2_^−^), hydroxyl radicals (·OH), and holes (h^+^), respectively, and the obtained results were compared with those of a control group without any scavenger addition [38].

### 2.8. EPR

Photogenerated ROS were detected using an electron paramagnetic resonance (EPR) spectrometer (Bruker EMXplus-6/1, Bruker, Karlsruhe, Germany). First, the SF/PAgT-4 fiber membranes were subjected to ultrasonic lysis in deionized water or methanol. Then, 2,2,6,6-Tetramethylpiperidin-1-oxyl (TEMPO) and 5,5-dimethyl-1-pyrroline N-oxide (DMPO) were used as spin trapping reagents. After thorough homogenization, the mixture was transferred into the EPR resonator to characterize photogenerated free radicals.

## 3. Results and Discussion

### 3.1. Morphology and Structure Analysis

**Figure 1:**
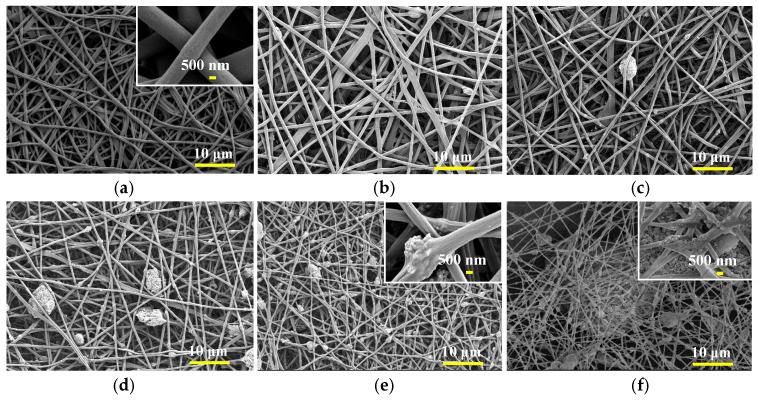
SEM images of different fiber membranes: (**a**) SF; (**b**) SF/PAgT-1; (**c**) SF/PAgT-2; (**d**) SF/PAgT-3; (**e**) SF/PAgT-4 and (**f**) SF/PAgT-5.

**Figure 2:**
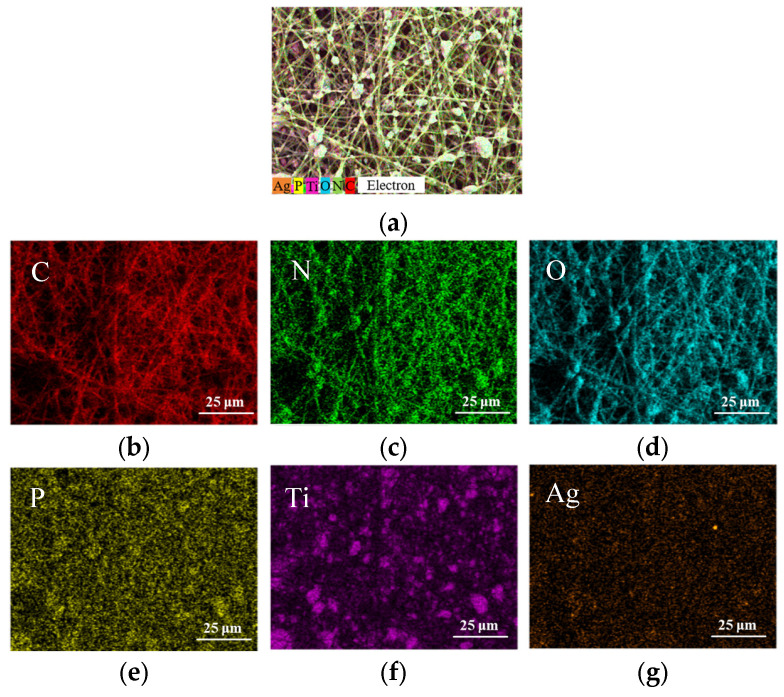
EDX images of (**a**) selected area, (**b**) C, (**c**) N, (**d**) O, (**e**) P, (**f**) Ti, (**g**) Ag of SF/PAgT-4 fiber membrane.

At first, the morphology of the as-prepared SF/PAgT photocatalytic fiber membranes was characterized using SEM. It could be seen from Figure 1a that the pure SF fiber had a uniform diameter and a smooth surface morphology. The pristine PAgT photocatalyst powder demonstrated irregular rock topography (Figure S2). As shown in Figure 1b–f, the addition of a PAgT photocatalyst significantly affected the morphology of the SF fiber membrane. With the mass concentration of PAgT increasing, the diameter of fibers decreased gradually. This change was mainly ascribed to the enhanced conductivity of the spinning solution after the addition of the PAgT photocatalyst. During the electrospinning process, the addition of PAgT to the SF solution resulted in an increase in the charge density on the surface of the polymer jet, which led to a stronger tensile force and the formation of finer fibers. On the other hand, with the addition of PAgT to the SF solution, the surface of the SF/PAgT composite membrane showed protrusions and a spindle-shaped structure. Moreover, as the mass concentration of PAgT increased, the appearance of the spindle-shaped structure became more remarkable. However, when the concentration of the photocatalyst exceeded 0.20 g/mL (as shown in Figure 1f), the electrospinning performance deteriorated significantly. During the spinning process, it was observed that the jet containing the SF/PAgT-5 spinning solution was frequently interrupted, and the spinning nozzle repeatedly became clogged. This resulted in an extremely uneven membrane surface of the prepared SF/PAgT-5 sample, thereby hindering subsequent experimental and characterization work. For this reason, the SF/PAgT-5 membrane was excluded from subsequent tests. In order to investigate the element composition and distribution characteristics of the photocatalyst fiber membrane, the EDS elemental analysis diagrams of SF/PAgT-4 were conducted (Figure 2). The analysis result indicated that the SF/PAgT-4 fiber membrane not only contained the inherent C, O and N of SF, but also the P, Ag and Ti unique to PAgT, which were uniformly distributed on the fiber surface.

### 3.2. Structure Analysis

**Figure 3:**
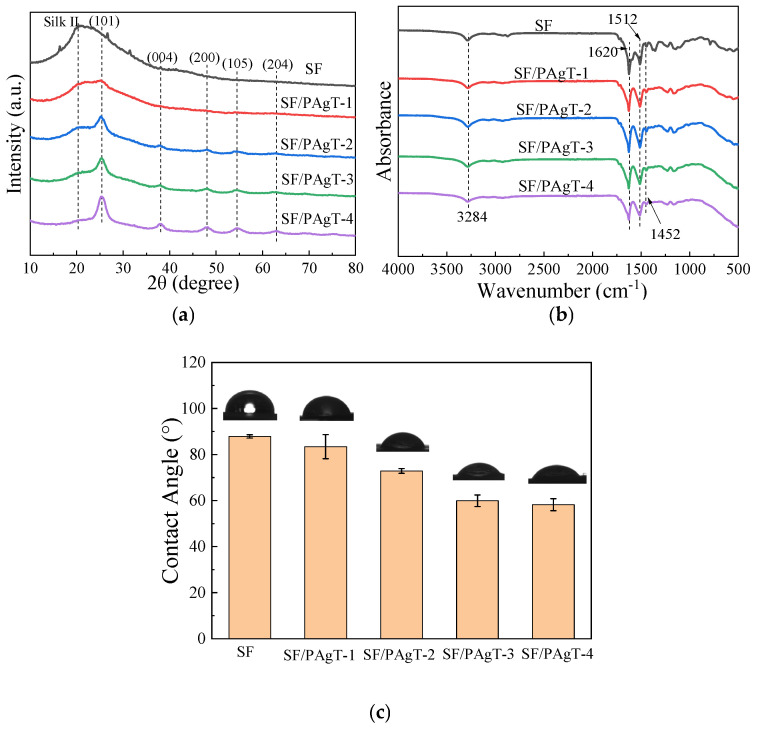
(**a**) XRD patterns, (**b**) FT-IR patterns and (**c**) Average CA values and representative droplets images of different fiber membranes.

The crystalline phase of the prepared fiber membranes was investigated by XRD patterns (Figure 3a). It can be found that the pure SF membrane generally exhibited a weak and broad hump, which is attributed to the amorphous structure of SF [35]. The peak located at 20.3° reflected that the primary structure of the as-prepared SF membrane was the more stable Silk II type. Upon the incorporation of the PAgT photocatalyst into the fiber membranes, the characteristic peaks at 25.4°, 37.9°, 48.0°, 54.4°, and 62.9° were observed in all SF/PAgT membranes, corresponding to the (101), (004), (200), (105), and (204) crystal planes of the TiO_2_ anatase phase, respectively [36]. This result indicated that the PAgT had been loaded onto the SF membrane. With the increase in the mass concentration of PAgT from SF/PAgT-1 to SF/PAgT-4, the intensity of the major diffraction peaks of TiO_2_ at 2θ of 25.4°, which belonged to the PAgT photocatalyst, was significantly enhanced. On the contrary, the diffraction signal attributed to SF gradually weakened. Obviously, the SF/PAgT-4 fiber membrane exhibited the strongest diffraction peaks of anatase TiO_2_ crystal structure. In addition, no peaks corresponding to Ag were detected by the XRD measurement, which could be attributed to the low concentration of Ag below the XRD detection limit. No crystalline phases or diffraction peaks corresponding to impurities were observed in any of the patterns, indicating the high purity of the prepared sample and the successful synthesis of the two-phase compound.

Figure 3b showed the FT-IR spectra of the as-prepared fiber membranes. Typically, within the absorption spectrum of silk fibroin, the region corresponding to the amide peaks (1700–1500 cm^−1^) serves as a reliable indicator of changes in its molecular conformation. The secondary structure of SF is primarily composed of Silk I and Silk II. The absorption peaks associated with the Silk I structure appear at 1650–1655 cm^−1^ (Amide I) and 1540–1555 cm^−1^ (Amide II), whereas those associated with the Silk II structure appear at 1610–1630 cm^−1^ (Amide I) and 1510–1520 cm^−1^ (Amide II). As shown in Figure 3b, the as-prepared SF material exhibited absorption peaks near the 1620 and 1512 cm^−1^ band, indicating that the primary structure of the as-prepared SF membrane was Silk II type. This was mainly because during the preparation of electrospinning SF material, treatment with organic solvent can induce the structural transformation of fibroin from the disordered Silk I form to the stable Silk II form (β-sheet). In addition, the SF fiber membrane exhibited a distinct absorption peak at approximately 3284 cm^−1^, which was attributed to O-H and N-H stretching vibrations, whereas the absorption peak at 1452 cm^−1^ was associated with C-N stretching vibration. More importantly, following the addition of PAgT, the characteristic peaks of SF remained observable in all SF-PAgT samples, and the positions of their absorption peaks exhibited virtually no change. This result indicated that the binding of SF with the PAgT photocatalyst did not disrupt its chemical structure and original properties. Nevertheless, the characteristic peaks belonging to the PAgT photocatalyst were not observed in all the SF/PAgT fiber membranes. This may be attributed to the relatively low content of PAgT in the prepared fiber membrane samples.

To investigate the hydrophilicity and hydrophobicity of the developed SF/PAgT fiber membranes with different PAgT contents, the CA measurements were conducted (Figure 3c). Based on the standard threshold (hydrophilic < 90° or hydrophobic > 90°), the interaction at the liquid–solid interface was quantified by measuring the angle of the water droplets deposited on the fiber membranes [39]. It can be found that the CA of the SF sample was approximately 87.9°, which indicated moderate hydrophilicity of the SF fiber membrane. This was primarily attributed to the abundant hydrophilic groups within the SF molecular chains. After the introduction of PAgT, as the mass concentration of PAgT increased, the CA of the fiber membranes gradually decreased. The CA of SF/PAgT-4 was only 58.2°, demonstrating significantly enhanced hydrophilicity. Those results could be attributed to the following reasons: one is that the hydroxyl groups (-OH) could readily form on the surface of TiO_2_ within the material and were capable of forming hydrogen bonds with water molecules; furthermore, the high surface energy of TiO_2_ facilitated the adsorption of water molecules, thereby endowing the SF/PAgT membrane with hydrophilic properties. This enhanced hydrophilicity of the developed SF/PAgT material could facilitate O_2_ adsorption, thereby promoting the photocatalytic generation of reactive oxygen species and further favoring the photocatalytic process.

### 3.3. Antibacterial Activity

**Figure 4:**
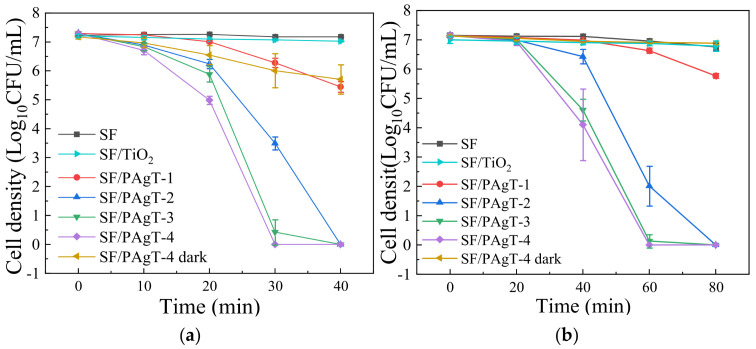
Photocatalytic inactivation effect of different fiber membranes on (**a**) *E. coli* and (**b**) *S. aureus* under visible light irradiation.

The inactivation efficiency of different prepared photocatalytic fiber membranes on *E. coli* and *S. aureus* is shown in Figure 4a and Figure 4b, respectively. Photos of the bacterial culture dishes during the photocatalytic inactivation experiment are shown in Figure S3. As shown in Figure 4a, the concentration of *E. coli* suspension remained essentially unchanged after 40 min of treatment with the pure SF and SF/TiO_2_ fiber membrane under visible light irradiation. This result confirmed that pure SF and SF/TiO_2_ fiber membranes did not possess antibacterial activity under visible light irradiation. After introducing PAgT photocatalyst to the SF fiber membrane, the photocatalytic antibacterial performance of the SF/PAgT composite fiber membranes improved. SF/PAgT-2 and SF/PAgT-3 were capable of completely inactivating 10^7^ CFU/mL of *E. coli* within 40 min of light irradiation. SF/PAgT-4 exhibited the most superior antibacterial performance, requiring only 30 min of light irradiation to completely inactivate 10^7^ CFU/mL of *E. coli*. The obtained result suggested that as the PAgT content increased, the photocatalytic antibacterial performance of the SF/PAgT composite fiber membranes was also enhanced. This was primarily attributed to the increased number of active sites on the catalyst, thereby leading to a continuous enhancement of the composite fiber membranes’ antibacterial properties. A similar sterilization tendency can also be observed against *S. aureus* (Figure 4b). SF/PAgT-4 can achieve a complete inactivation of 10^7^ CFU/mL *S. aureus* only within 60 min under visible light. Compared with *E. coli*, the inactivation efficiency of the composite fiber membrane against *S. aureus* was slightly lower, which was mainly due to the different cell wall structure of the two kinds of bacteria. *S. aureus*, as a Gram +ve bacterium, possesses a relatively thicker peptidoglycan layer within its cell wall, which can effectively prevent the penetration of reactive oxygen species, thereby rendering it more difficult to be inactivated [40]. The results indicated that the SF/PAgT-4 material exhibited optimal photocatalytic antibacterial performance, suggesting that the optimal doping mass concentration of PAgT during the synthesis process was 0.20 g/mL. Therefore, the SF/PAgT-4 material was selected for further catalytic studies as the most effective photocatalytic fiber membrane. Based on preliminary antibacterial assays, treatment durations of 30 min for *E. coli* and 60 min for *S. aureus* were selected to observe the colony-forming results. The photographs of *E. coli* (Figure S3a) and *S. aureus* (Figure S3b) culture plates further confirmed that after being treated by SF/PAgT-4 under light, all bacteria were completely inactivated. Moreover, a comparison of the photocatalytic disinfection efficiency by different reported photocatalytic membranes was further conducted and is summarized in Table S1. The disinfection efficiency of the SF/PAgT-4 photocatalytic fiber membrane in this study reached 1.25 × 10^6^ CFU/(cm^2^·min) for *E. coli* and 6.25 × 10^5^ CFU/(cm^2^·min) for *S. aureus*, which were higher than most reported photocatalysts in treating the same bacterial species [41,42,43,44,45,46].

**Figure 5:**
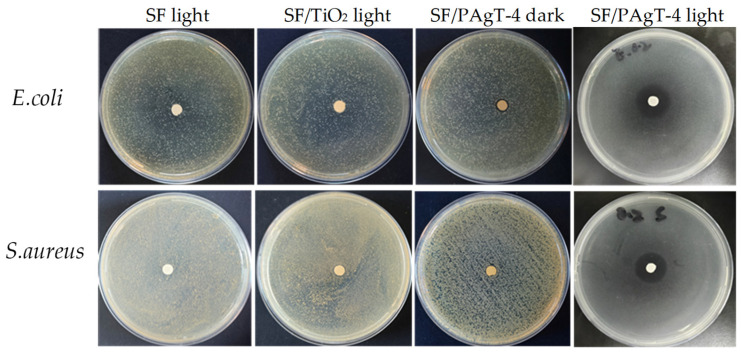
Inhibition zones against *E. coli* and *S. aureus* after treatment with different fiber membranes.

The antibacterial zone test of the developed SF/PAgT composite fiber membranes was also conducted, and the results are presented in Figure 5. The pure SF and SF/TiO_2_ fiber membrane did not show a significant inhibitory zone under visible light irradiation, which indicated that it did not possess antibacterial activity. Similarly, SF/PAgT-4 in the absence of light also did not show any significant inhibition zones against *E. coli* and *S. aureus*. In contrast, under visible light conditions, the inhibition zones of SF/PAgT-4 against *E. coli* and *S. aureus* reached 20 mm and 19 mm, respectively. Overall, compared with pure SF and SF/TiO_2_ fiber membranes, the developed SF/PAgT-4 fiber membranes had significantly enhanced inhibitory effects on the two types of bacteria.

### 3.4. Recyclable and Thermal Stability

**Figure 6:**
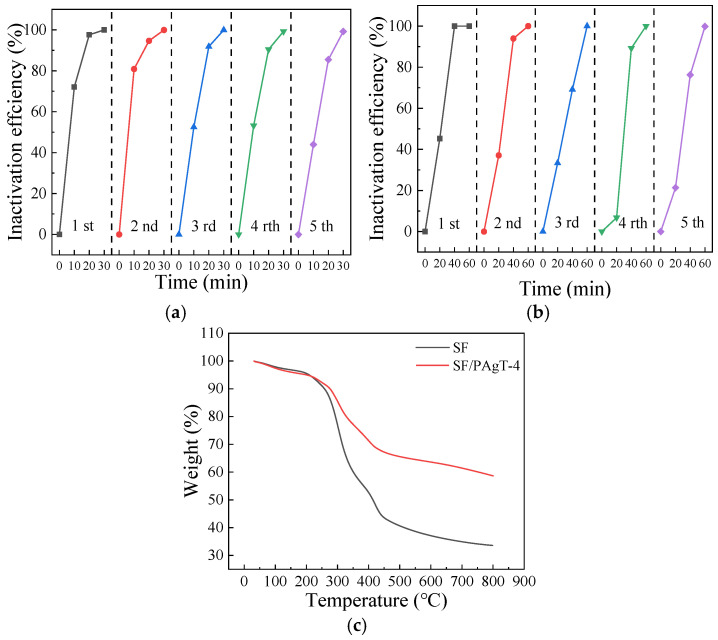
Repetitive photocatalytic inactivation performance of SF/PAgT-4 on (**a**) *E. coli* and (**b**) *S. aureus*; (**c**) TGA images of SF and SF/PAgT-4 fiber membranes.

The reusability and stability of a catalyst are key parameters for assessing its industrial feasibility to achieve the highest catalyst output. To evaluate this, recycling experiments and thermogravimetric analysis were conducted using the SF/PAgT-4 material under constant reaction conditions to monitor any changes in catalytic activity. As shown in Figure 6a,b, SF/PAgT-4 demonstrated excellent stability during five consecutive cycles of use. Although the inactivation rate of *E. coli* decreased in the fifth cycle, 99.2% inactivation could still be achieved within 30 min (Figure 6a). The inactivation against *S. aureus* also showed a similar tendency. As illustrated in Figure 6b, the inactivation efficiency remained essentially unchanged across all reuse cycles. After the fifth cycle, 99.8% *S. aureus* could still be inactivated within 60 min, indicating excellent catalyst stability and preservation of active sites.

Additionally, to evaluate the thermal stability of the developed fiber membranes, thermogravimetric analysis (TGA) was performed under a nitrogen atmosphere. Figure 6c demonstrated the TG curves of SF and SF/PAgT-4. Regarding the TG curve of the SF fiber membrane, the entire reaction process can be divided into three stages. The first stage, occurring between 30 and 100 °C, was attributed to the evaporation of water adsorbed on the membrane. In the second stage (100–250 °C), the membranes’ weight remained almost unchanged, indicating good thermal stability of the material within this temperature range. Subsequently, in the third stage (250–800 °C), the SF fiber membrane showed a weight loss of 66.4%. This was attributed to the fact that the secondary and tertiary structures of the SF were disrupted, the peptide bonds linking the amino acids were cleaved, and the fibroin was gradually degraded into organic carbon. As for the TG curve of SF/PAgT-4 material, it exhibited the same trend as that of SF. However, its weight loss during the third stage was lower than that of SF, with a weight loss rate of only 41.2%, which was attributed to the incorporation of PAgT into the SF/PAgT-4 photocatalytic fiber membrane. The primary component of PAgT was TiO_2_, which possessed excellent thermal stability and remained largely undecomposed at temperatures below 800 °C [47]. The aforementioned results indicated that the SF/PAgT-4 photocatalytic fiber membrane could maintain good thermal stability below 250 °C, sufficiently meeting the requirements for its practical application as a bactericidal material. Based on the cyclic experiment and TGA results, the SF/PAgT-4 fiber membrane not only demonstrated excellent recyclable stability but also possessed remarkable thermal stability, highlighting its potential for effective and sustainable utilization in practical water treatment applications.

### 3.5. Cell Membrane Injury

**Figure 7:**
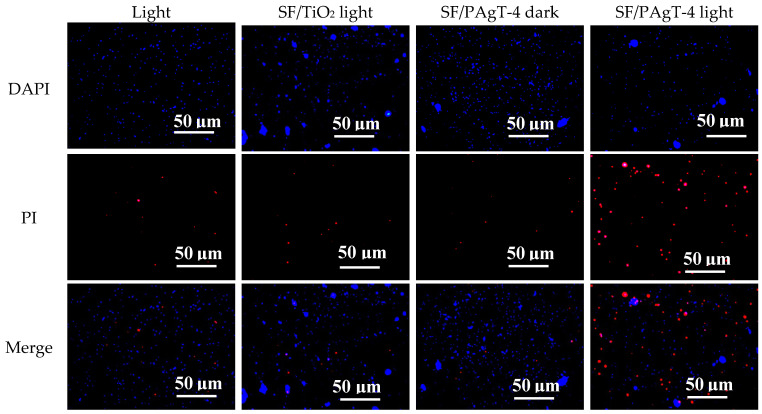
Fluorescent images of the DAPI-PI double-stained *E. coli* after being treated by SF/PAgT-4 fiber membrane (The PI-positive (red) cells indicated the dead cells, and the DAPI-positive (blue) cells indicated bacterial DNA (nucleoids)).

**Figure 8:**
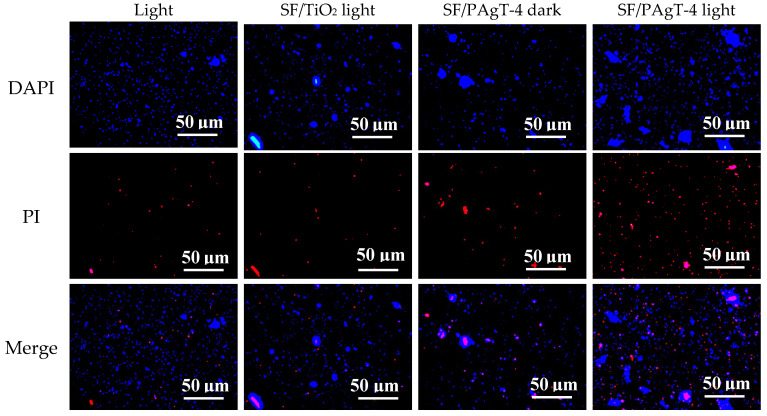
Fluorescent images of the DAPI-PI double-stained *S. aureus* after treatment by SF/PAgT-4 fiber membrane (The PI-positive (red) cells indicated the dead cells, and the DAPI-positive (blue) cells indicated bacterial DNA (nucleoids)).

To demonstrate the permeability of bacterial cell membranes during the photocatalytic treatment process, bacteria cells were double-stained with DAPI and PI. DAPI was a fluorescent dye that could freely penetrate the cell membrane to bind to double-stranded DNA in the nucleus and could be excited by UV radiation to produce blue fluorescence regardless of whether the cell was intact or not, which could be used to label all bacteria. In contrast, PI could only penetrate damaged cell membranes and then bind to nucleic acids to produce red fluorescence, thus verifying cell activity and membrane permeability. As shown in Figure 7 and Figure 8, in the control groups of light irradiation alone, SF/TiO_2_ with light and SF/PAgT-4 without light irradiation, a significant quantity of blue fluorescence and a negligible amount of red fluorescence were observed for both *E. coli* and *S. aureus*. The small number of dead bacteria in the control group may be due to natural bacterial death during the culture process. However, after treatment with the SF/PAgT-4 photocatalytic fiber membrane under light irradiation, a significant increase in red fluorescence could be observed for both *E. coli* (Figure 7) and *S. aureus* (Figure 8), which indicated that the cell membrane of *E. coli* and *S. aureus* had been damaged under this condition. In addition, as illustrated in Figure S4, the protein concentration in the reaction solution gradually increased with prolonged treatment time of *E. coli* and *S. aureus* by SF/PAgT-4 membranes. Those were in line with the results obtained from the previous plate counting (Figure 4), which indicated that the SF/PAgT-4 fiber membrane was capable of causing cell membrane injury, thereby leading to the intracellular component leakage and inducing extensive bacterial inactivation.

### 3.6. Mechanisms of Bacteria Inactivation

**Figure 9:**
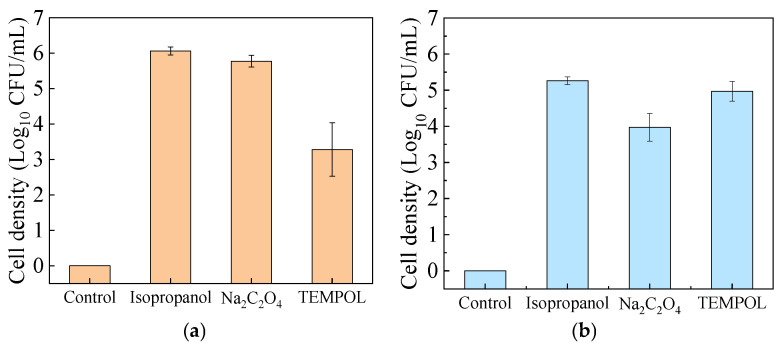
Photocatalytic disinfection of (**a**) *E. coli* and (**b**) *S. aureus* by SF/PAgT-4 fiber membrane in the presence of different scavengers (inactivation time: *E. coli* was 30 min and *S. aureus* was 60 min; control: no scavenger addition).

To further clarify the contribution of various ROSs during the photocatalytic disinfection process by SF/PAgT-4, the study on scavengers was carried out (Figure 9). Compared to the bacterial inactivation situation without the addition of the clearing agent, the addition of isopropanol, Na_2_C_2_O_4_ and TEMPOL inhibited the inactivation process of *E. coli* and *S. aureus* (Figure 9), proving that all three radicals, including ·OH, h^+^ and ·O_2_^−^, participated in the bactericidal process of the SF/PAgT-4 material. Furthermore, in order to confirm the production of ROS by SF/PAgT-4, the EPR experiments were conducted in the presence and absence of visible light. As displayed in Figure S5a,b, no characteristic signals corresponding to ·OH and ·O_2_^−^ were detected under dark conditions. In contrast, the typical four-line characteristic peak assigned to ·OH emerged upon visible light irradiation, accompanied by the distinct characteristic peak of ·O_2_^−^. These results verify that both ·OH and ·O_2_^−^ reactive oxygen species can be produced over SF/PAgT-4 fiber membrane upon visible light illumination. The reduction in characteristic peak intensities in Figure S5c confirms the formation of h^+^. Those results indicated that various radicals (·OH, ·O_2_^−^ and h^+^) were generated by SF/PAgT-4 under visible light, which can lead to the death of bacteria.

**Figure 10:**
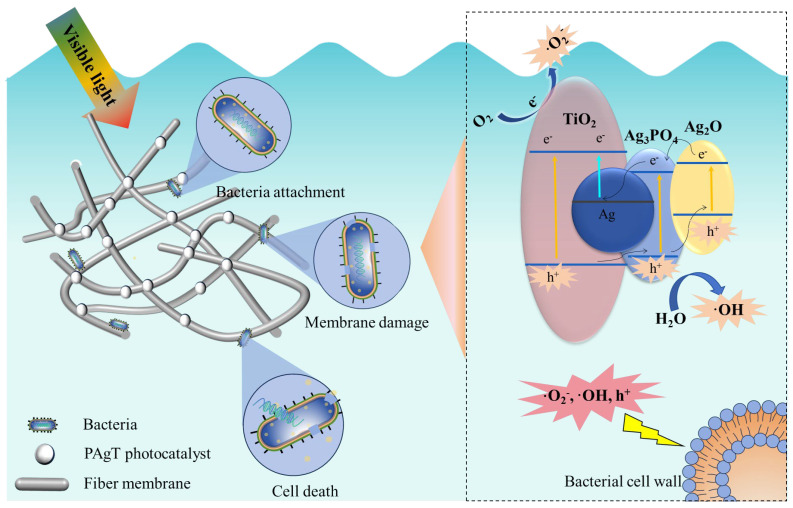
Proposed mechanism illustration of SF/PAgT-4 fiber membrane for efficient bacterial disinfection under visible light irradiation.

Based on the aforementioned results, the possible photocatalytic antibacterial mechanism of an SF/PAgT-4 fiber membrane was proposed. Figure 10 presents the specific process of photocatalytic bacterial inactivation. When the SF/PAgT-4 fiber membrane was placed in the bacterial solution, bacterial cells adhered to the surface of the fiber membrane. After exposure to visible light, the photocatalytic process was immediately initiated, assuming the role of bacterial disinfection. The possible pathways for generating active species had been proposed based on the previous report [37]. In detail, under light excitation, the SF/PAgT-4 fiber membrane generated electrons (e^−^) and holes (h^+^). Specifically, the electrons were efficiently captured at the less negative Ag and subsequently transferred to the TiO_2_ surface owing to the surface plasmon resonance (SPR) effect, which could then combine with adsorbed O_2_ to generate superoxide radicals (·O_2_^−^) [48]. Accordingly, the produced h^+^ moved to Ag_2_O following the transfer principle from a higher energy level to a lower energy level. Meanwhile, hydroxyl radicals (·OH) can be formed when photogenerated h^+^ were captured by H_2_O [49]. Subsequently, all of these photogenerated radicals (·O_2_^−^, ·OH and h^+^) attacked the bacterial cells, causing the cell membrane to be damaged, further destroying the intracellular proteins, DNA and other biomolecules, and promoting bacterial inactivation. It provides a possible solution for the effective control of waterborne bacterial pollutants and expands a new development direction for water purification technology. The current bottleneck regarding the difficult recovery and reuse of powdered photocatalytic materials will be effectively resolved.

## 4. Conclusions

In summary, a novel SF/PAgT fiber membrane was successfully developed by incorporating PAgT photocatalyst powder into the SF fiber membrane via a facile electrospinning method in this work. During the synthesis process, the mass concentration of the PAgT dopant was optimized, and the results demonstrated that SF/PAgT-4 exhibited significant antibacterial activity against both *E. coli* and *S. aureus* under visible light irradiation, indicating 0.20 g/mL was the optimal doping mass concentration of PAgT. The cyclic experiment and thermogravimetric analysis clarified that the SF/PAgT-4 fiber membrane holds excellent recyclability and thermal stability, highlighting its potential for sustainable utilization for antibacterial applications. Furthermore, it was found that during the current photocatalytic disinfection system by SF/PAgT-4 material, h^+^, ·O_2_^−^ and ·OH played crucial roles and were capable of attacking the bacterial cells, causing the cell membrane injury, thereby inducing extensive bacterial inactivation. Hence, by virtue of its ease of recovery, excellent thermal stability, and superior antimicrobial activity, the developed SF/PAgT-4 fiber membrane holds immense potential for efficient and sustainable utilization. The findings of this study will contribute to advancing the application of powdered photocatalysts in practical large-scale wastewater treatment. The current work mainly focuses on the preparation of materials and the assessment of photocatalytic activity. However, no in-depth exploration has been conducted on the mechanical strength and flexibility of the fiber membranes. Moreover, the application of these fiber membranes in actual wastewater treatment is still at a theoretical stage. In the future, the mechanical properties of the fiber membranes and their effects on actual wastewater treatment will be further investigated.

## Data Availability

The original contributions presented in this study are included in the article/Supplementary Materials. Further inquiries can be directed to the corresponding author.

## Supplementary Materials

The following supporting information can be downloaded at: https://www.mdpi.com/article/10.3390/polym18131632/s1, Figure S1: Schematic representation of the cyclic experiments. Figure S2: SEM image of pristine PAgT photocatalyst powder. Figure S3: The bacterial plate images of (a) *E. coli* and (b) *S. aureus* after treated with different fiber membranes for 30 min and 60 min respectively. Figure S4: Protein leakage from (a) *E. coli* and (b) *S. aureus* after treated by SF/PAgT-4 fiber membrane under visible light irradiation (Light control: no SF/PAgT-4 fiber membrane addition). Figure S5: EPR spectra of (a) ⋅OH, (b) ⋅O_2_^−^ and (c) h^+^ under visible illumination and dark conditions. Table S1: Comparation of different reported photocatalytic membranes disinfection performance towards different target microorganisms.
