# Supplementary material for: Electrospinning Preparation of Silk Fibroin/Titanium-Based Photocatalytic Fiber Membrane for Bacteria Disinfection in Wastewater
Source: Polymers (Basel). 2026 Jun 30;18(13):1632. doi: 10.3390/polym18131632 (PMC13363720; doi:10.3390/polym18131632)
Supplement: Supplementary file 1 [file polymers-18-01632-s001.zip › polymers-4392522-supplementary.pdf]

Supplementary Material

Electrospinning preparation of silk fibroin/titanium-based photocatalytic fiber membrane for bacteria disinfection in wastewater

Kuo Wang [1], Xiaoxuan Liu [1], Dading Zhou [1], Yujun Wang [2], Qiansu Ma [3], Yingnan Yang [4] and Na Liu [2,*]

[1] Department of Biomedical Engineering; University of Chengde Medical; Chengde 067000; China; 13293245945@163.com (K.W.); heee2782@163.com (X.L.); 18353236072@163.com (D.Z.)

[2] Department of Biological and Food Sciences; University of Chengde Medical; Chengde 067000; China; wangyj@cdmc.edu.cn (Y.W.)

[3] School of Chemistry and Biological Engineering; University of Science and Technology Beijing; Beijing 100083; China; qiansuma@ustb.edu.cn (Q.M.)

[4] Graduate School of Life and Environmental Sciences; University of Tsukuba; 1-1-1 Tennoudai; Tsukuba; Ibaraki; 305-8577; Japan; yo.innan.fu@u.tsukuba.ac.jp (Y.Y.)

[*] Correspondence: naliu66@cdmc.edu.cn (N.L.); Tel.: +86-13103045772

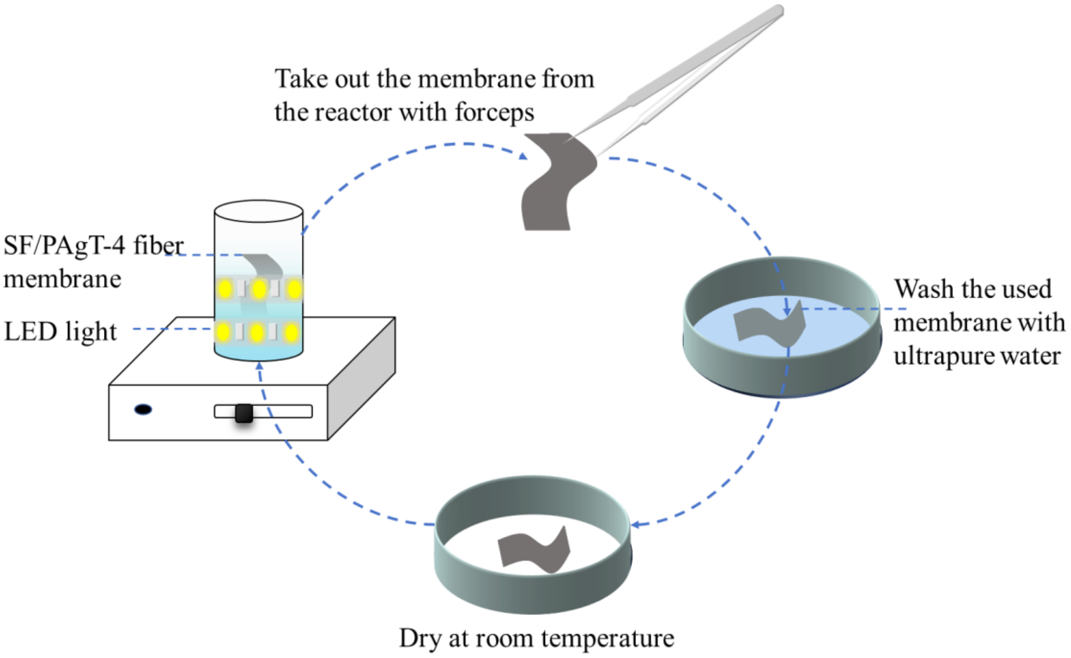

Figure S1. Schematic representation of the cyclic experiments.

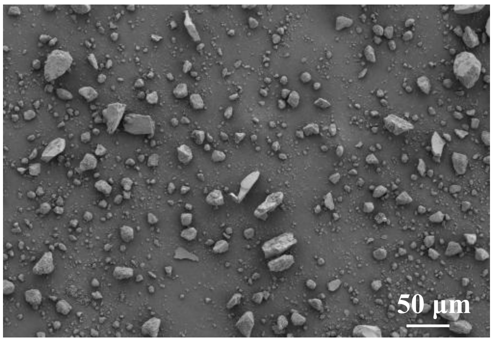

Figure S2. SEM image of pristine PAgT photocatalyst powder.

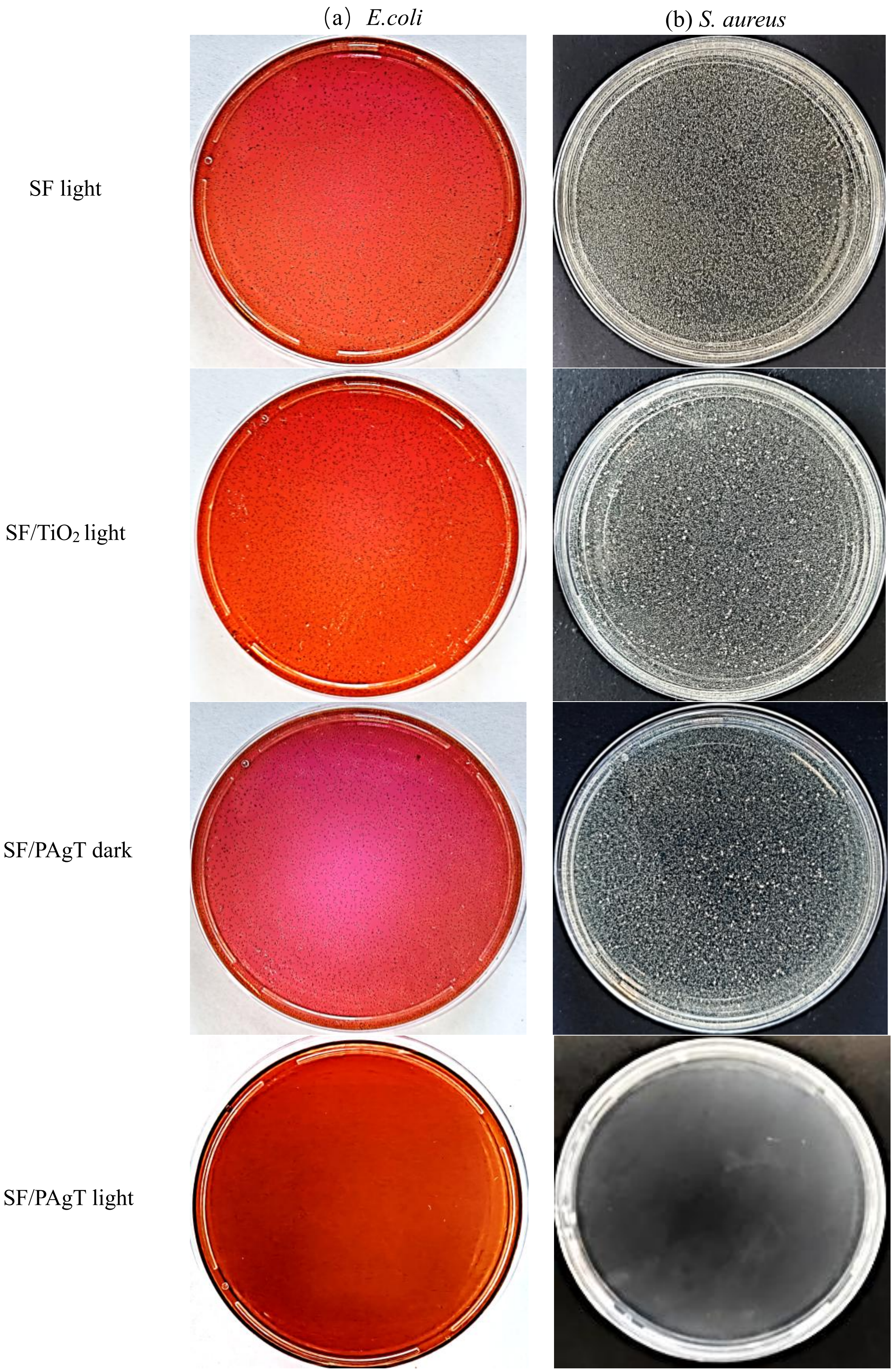

Figure S3. The bacterial plate images of (a) *E. coli* and (b) *S. aureus* after treated with different fiber membranes for 30 min and 60 min respectively.

The experiment procedure for protein concentration analysis

Total protein concentration was determined using a Bicinchoninic Acid Protein (BCA) Assay Kit (Solarbio, Beijing, China). The bacterial suspensions were treated with SF/PAgT-4 following the experimental procedure described in Section 2.5. The harvested bacterial suspensions were centrifuged (12000 rpm, 5 min). The resulting supernatant was mixed thoroughly with freshly prepared BCA working solution. The BCA working solution was prepared by mixing BCA and Cu at 50:1. The mixture was incubated for 30 min, and the absorbance was measured at 562 nm.

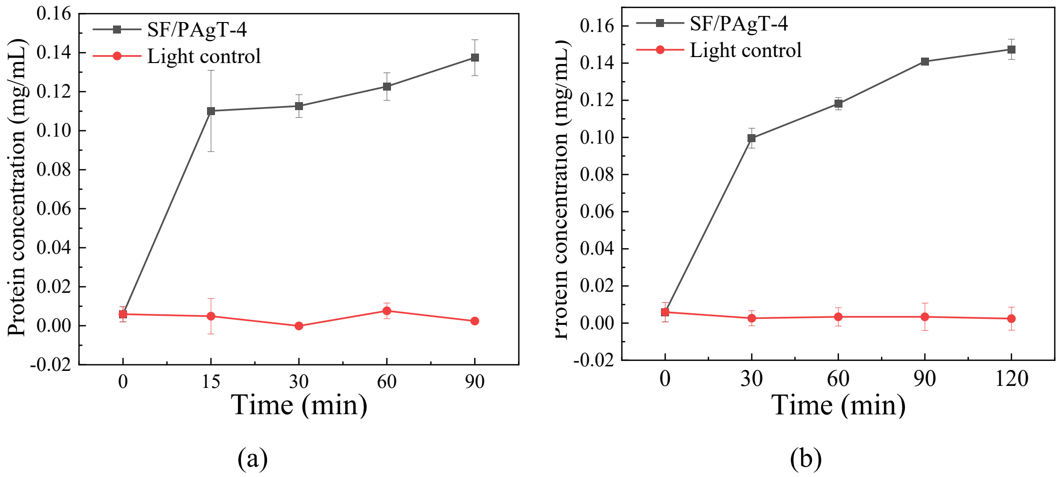

Figure S4. Protein leakage from (a) *E. coli* and (b) *S. aureus* after treated by SF/PAgT-4 fiber membrane under visible light irradiation (Light control: no SF/PAgT-4 fiber membrane addition).

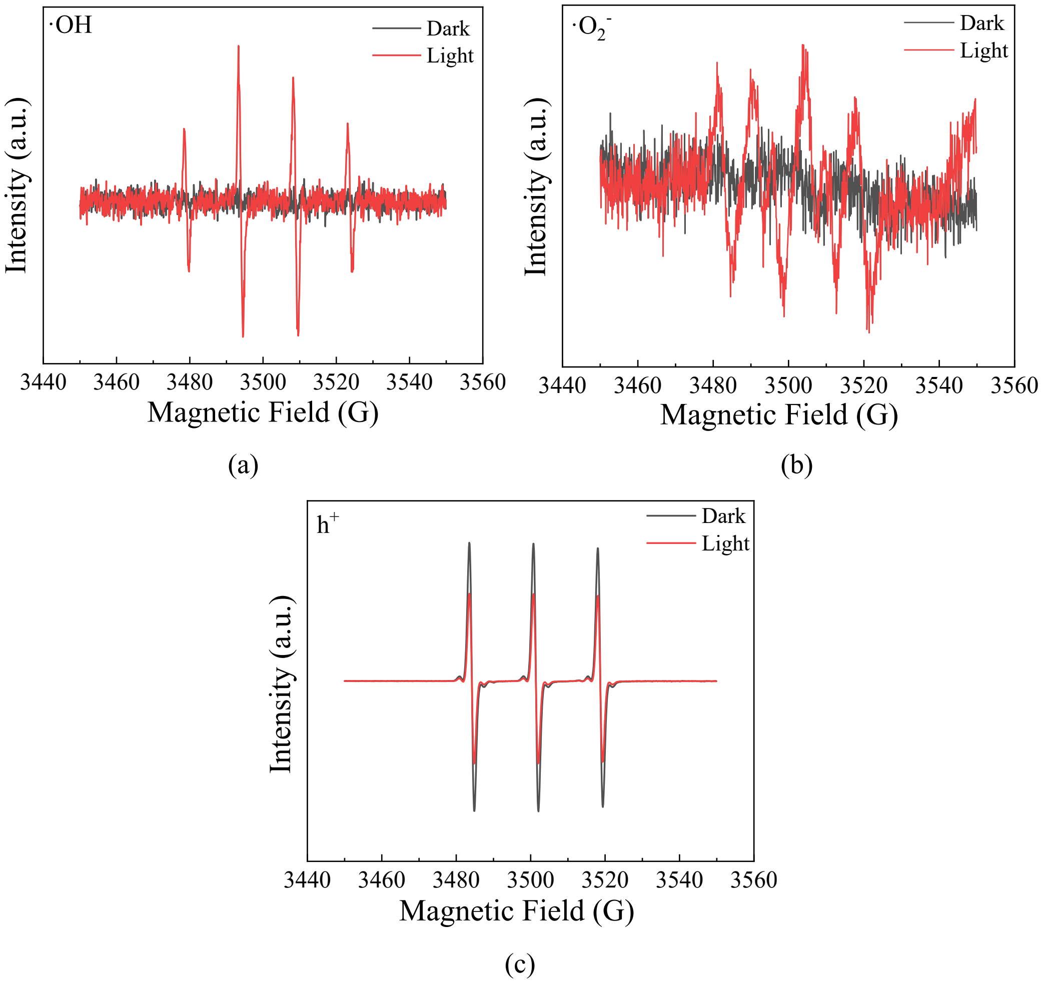

Figure S5. EPR spectra of (a) · OH, (b) · O_2^- and (c) h^+ under visible illumination and dark conditions.

Table S1 Comparation of different reported photocatalytic membranes disinfection performance towards different target microorganisms.

Target microorganisms	Photocatalyst	Bacterial reduction (CFU/mL)	Reaction volume (mL)	Membrane area (cm^2)	Inactivation time (min)	Inactivation efficiency (CFU/(cm^2·min))	Reference
E. coli	SF/PAgT-4 (LED visible light)	10^7	15	4.00	30	1.25×10^6	This study
E. coli	carbon/ZIF-67@PAN (xenon lamp)	10^5	1	3.14	20	1.59×10^3	[41]
E. coli	PP/PAN$_{30\%\ PDA}$ (NIR)	2×10^6	2	0.25	15	1.07×10^6	[42]
E. coli	TiO_2-ZnO (visible light)	9.8×10^4	10	1.00	60	1.63×10^4	[43]
S. aureus	SF/PAgT-4 (LED visible light)	10^7	15	4.00	60	6.25×10^5	This study
S. aureus	PpIX/CA (Xe lamp)	10^8	0.1	12	30	2.78×10^4	[44]
S. aureus	TiO_2 film catalyst (solar-simulated radiations)	6×10^6	100	37.5	180	3.17×10^5	[45]
S. aureus	PLA-0.2 Ce-N-TiO_2 (xenon lamp)	10^6	0.1	0.36	30	9.26×10^3	[46]
